# Phylogenetics of subtribe Orchidinae s.l. (Orchidaceae; Orchidoideae) based on seven markers (plastid *matK*, *psaB, rbcL, trnL-F, trnH-psba,* and nuclear nrITS, *Xdh*): implications for generic delimitation

**DOI:** 10.1186/s12870-017-1160-x

**Published:** 2017-11-25

**Authors:** Wei-Tao Jin, André Schuiteman, Mark W. Chase, Jian-Wu Li, Shih-Wen Chung, Tian-Chuan Hsu, Xiao-Hua Jin

**Affiliations:** 1 State Key Laboratory of Systematic and Evolutionary Botany, Institute of Botany, Chinese Academy of Sciences, Beijing, 10093 China; 2 Identification and Naming Department, Royal Botanic Gardens, Kew, Richmond, Surrey TW9 3AB UK; 3 Jodrell Laboratory, Royal Botanic Gardens, Kew, Richmond, Surrey TW9 3DS UK; 4 School of Plant Biology, University of Western Australia, Crawley, Perth, 6009 Australia; 5 Xishuangbanna Tropical Botanical Garden, Chinese Academy of Sciences, Menglun Township, Mengla County, Yunnan 666303 China; 6 Botanical Garden Division, Taiwan Forestry Research Institute, 53 Nanhai Road, Taipei, Taiwan 10066 China; 7 Department of Molecular and Cellular Biology, National Tsing Hua University, Hsinchu, Taiwan 30013 China; 8 Southeast Asia Biodiversity Research Institute, Chinese Academy of Science (CAS-SEABRI), Yezin, Nay Pyi Taw, Myanmar

**Keywords:** *Gennaria*, *Habenaria*, Orchidinae s.l., Orchid generic delimitation, *Ponerorchis* alliance, *Platanthera*

## Abstract

**Background:**

Subtribe Orchidinae (Orchidaceae, Orchidoideae) are a nearly cosmopolitan taxon of terrestrial orchids, comprising about 1800 species in 47 to 60 genera. Although much progress has been made in recent years of phylogenetics of Orchidinae, considerable problems remain to be addressed. Based on molecular phylogenetics, we attempt to illustrate the phylogenetic relationships and discuss generic delimitation within Orchidinae. Seven DNA markers (five plastid and two nuclear), a broad sampling of Orchidinae (400 species in 52 genera) and three methods of phylogenetic analysis (maximum likelihood, maximum parsimony and Bayesian inference) were used.

**Results:**

Orchidinae s.l. are monophyletic. *Satyrium* is sister to the rest of Orchidinae s.l. *Brachycorythis* and *Schizochilus* are successive sister to Asian-European Orchidinae s.s. *Sirindhornia* and *Shizhenia* are successive sister to clade formed by *Tsaiorchis*-*Hemipilia*-*Ponerorchis* alliance. *Stenoglottis* is sister to the *Habenaria-Herminium-Peristylus* alliance. *Habenaria*, currently the largest genus in Orchidinae, is polyphyletic and split into two distant clades: one Asian-Australian and the other African–American–Asian. *Diplomeris* is sister to *Herminium* s.l. plus Asian-Australian *Habenaria*.

**Conclusions:**

We propose to recognize five genera in the *Ponerorchis* alliance: *Hemipilia*, *Ponerorchis* s.l., *Sirindhornia*, *Shizhenia* and *Tsaiorchis*. Splitting *Habenaria* into two genera based on morphological characters and geographical distribution may be the least disruptive approach, and it is reasonable to keep *Satyrium* in Orchidinae.

**Electronic supplementary material:**

The online version of this article (10.1186/s12870-017-1160-x) contains supplementary material, which is available to authorized users.

## Background

**Fig. 1:**
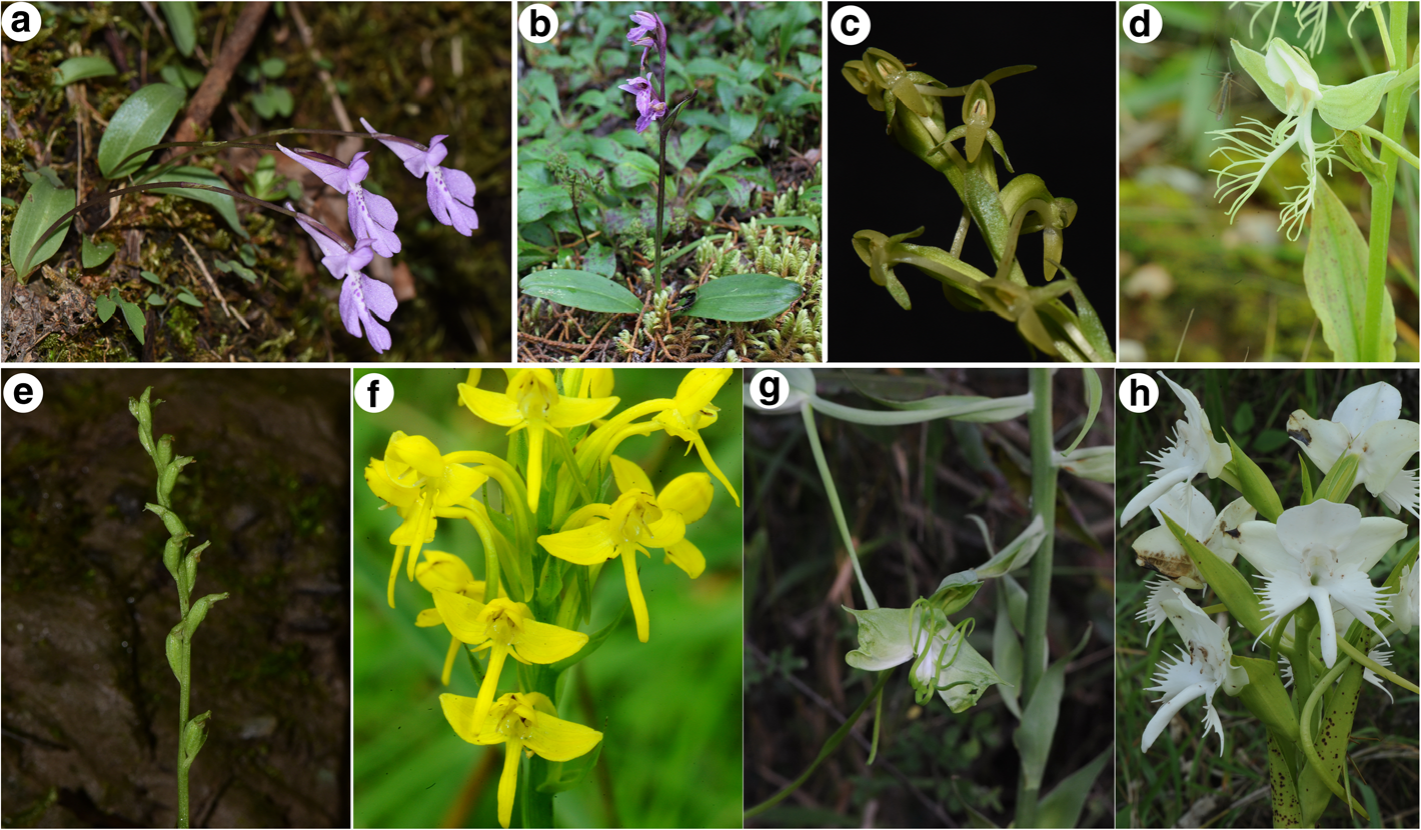
Representative species of Orchidinae*.* **a**, *Shizhenia pinguicula;* **b**, *Galearis spathulata*; **c**, *Platanthera bakeriana*; **d**, *Habenaria davidii*; **e**, *Gennaria griffthii*; **f**, *Habenaria linguella*; **g**, *Bonatea steudneri*; **h**. *Pecteilis susannae*. (a by Weitao Jin, g by Bing Liu, others by Xiaohua Jin)

Subtribe Orchidinae are a nearly cosmopolitan clade of mostly terrestrial orchids, comprising about 1800 species in 47 to 60 genera [1, 2]. Morphologically, Orchidinae are characterized by: fleshy roots or tubers; non-plicate, convolute leaves that are basal or spirally arranged along the stem (Fig. 1); terminal, racemose inflorescences carrying mostly resupinate, small flowers (predominantly white, green or purple; Fig. 1); a single, usually erect anther lacking an operculum; two sectile pollinia; a column with two lateral appendages; stigma appearing 2–3-lobed (Fig. 1) [2–4]. Various genera within Orchidinae have been the subject of recent systematic studies, including *Dactylorhiza* [5, 6]; *Gymnadenia* [7, 8]; *Habenaria* [9]; *Herminium* [10]; *Himantoglossum* [11]; *Ophrys* [12–14]; *Orchis* [15–17]; *Platanthera* [18–22]; and *Ponerorchis* [23, 24]. These studies inevitably addressed only part of the diversity of the subtribe as a whole.

More comprehensive systematic investigations of Orchidinae have elucidated certain parts of the clade and also indicated areas where our knowledge is still inadequate [25–27]. Using nuclear ribosomal ITS (nrITS) from 190 species, Bateman et al. [26] identified 12 well-resolved clades within Orchidinae, confirming triphyly of *Orchis* as traditionally delimited, polyphyly of *Habenaria*, and considerable levels of homoplasy of various morphological characters. Most of these findings have been confirmed by subsequent results [9, 19, 23–25]. The first study with a good selection of Asian taxa [23], based on three markers (plastid *matK*/*rbcL* and nrITS) from 146 species, found that several genera as traditionally delimited were not monophyletic. These authors combined *Hemipiliopsis* Y.B.Luo & S.C.Chen with *Hemipilia* Lindl., *Amitostigma* Schltr. and *Neottianthe* (Rchb.) Schltr. with *Ponerorchis*, *Smithorchis* Tang & F.T.Wang with *Platanthera*, and *Aceratorchis* Schltr. and *Neolindleya* Kraenzl. with *Galearis* Raf. (*Galearis*, Fig. 1b), and proposed the new genus *Hsenhsua* X.H.Jin, Schuit. & W.T.Jin to accommodate *Ponerorchis chrysea* (W.W.Sm.) Sóo. Tang et al. analysed the *Amitostigma/Ponerorchis/Hemipilia* alliance using a wider sampling of this group than previous studies and concluded that still broader generic limits in this alliance were appropriate. They combined *Ponerorchis*, *Hemipilia* and related genera into a single genus, applying the oldest available name, *Hemipilia*.

These results, together with the above-mentioned studies on particular genera of Orchidinae, have greatly improved our understanding of phylogenetics of the subtribe, although some controversy remains in the *Ponerorchis*/*Hemipilia* alliance, as we will demonstrate. With about 835 species [1], *Habenaria* is one of the largest genera in the Orchidaceae; it is widespread throughout the tropical and subtropical regions of the world [2]. Currently, it undoubtedly presents the biggest issue in Orchidinae phylogenetics [1]. Generic delimitation and infrageneric classification of *Habenaria* have been much debated [2, 28–30]. Recent molecular studies found *Habenaria* to be para- or polyphyletic [9, 19, 23, 25, 26]. The most extensive phylogenetic analysis of *Habenaria* published so far, Batista et al. [9], focused on the South American representatives and found these to form a clade nested within a larger group of African taxa.

In the present study, phylogenetic relationships were inferred using seven DNA markers (five plastid and two nuclear regions) based on a much broader sampling (approximately 52 genera and 400 species) across Orchidinae, especially for those undersampled or unrepresented in previous studies, with the aims of: (1) reconstructing the phylogenetic interrelationships within Orchidinae; and (2) rationalizing generic delimitation within Orchidinae in accordance with a phylogenetic framework. Of the genera recognized as members of Orchidinae [1], we were unable to obtain material of the following: *Bartholinia, Centrostigma, Dracomonticola, Holothrix, Megalorchis, Neobolusia, Oligophyton, Platycoryne, Roeperocharis, Silvorchis, Thulinia* and *Veyretella*. *Shizhenia* was proposed after publication of Chase et al. [1], and we have included it.

## Results

### Sequences and alignment

In total, 2474 sequences were included, of which 1294 were newly produced for this paper. The number of missing sequences in the combined matrix of all markers is 767. The characteristics of maximum parsimony analyses of each nuclear gene, combined nuclear genes, combined plastid genes and combined nuclear and plastid datasets are summarized in Additional file 6: Table S4.

### Phylogenetic inference

Based on the combined nuclear nrITS, *Xdh* and plastid DNA data, our findings are consistent in overall topology with the trees produced using ML, MP and BI methods, except for a few of the terminal nodes.

### Phylogeny inferred from plastid and nuclear data

The maximum parsimony analysis of the plastid data produced 375 MPTs with a length of 8819 steps, consistency index (CI) of 0.44, and retention index (RI) of 0.82. The strict consensus tree shows that most of the ingroup clades form a polytomy with *Satyrium* sister to the rest but with low support (Additional file 1: Figure S1). On the other hand, the ML tree and BI trees inferred from plastid data are highly resolved (Additional file 1: Figure S1). Nodes of the trees with BS_ML_/ BS_MP_ > 50 are considered weakly supported, > 70 as moderately supported, and > 80/85 and Bayesian inference (BI) 0.95 as strongly supported.

The maximum parsimony analysis of nrITS (including nrITS of the conflicting species) retrieved 75 MPTs of 7352 steps, consistency index of 0.20, and retention index of 0.79. The strict consensus tree (Additional file 2: Figure S2) includes ten major clades, the relationships of which are not well resolved: (1) 110 species of the *Platanthera-Galearis-Dactylorhiza-Gymnadenia-Orchis-Ophrys-Himantoglossum* alliance (about 20 genera) with high support (BS_ML_ = 100, BS_MP_ = 100, PP = 1.0; in this same order in the following results and discussion); (2) 65 species of *Ponerorchis-Hemipilia-Tsaiorchis-Shizhenia-Sirindhornia* (five genera) with moderate to weak support (69, 55, 0.74), within which six species (including *Ponerorchis graminifolia*) are sister to *Hemipilia* plus the remaining species of *Ponerorchis*; (3) four species of *Brachycorythis* with high support (100, 100, 1.0); (4) 100 species of the *Herminium-Habenaria* (p.p.) alliance (often considered to be four genera) with high to moderate support (92, 77, 0.99); (5) 19 species of *Peristylus* with high support (100, 100, 1.00)*;* (6) a clade of 26 species *Habenaria* (including the type) with high support (100, 100, 1); (7) seven species of *Bonatea-Habenaria* (p.p.) with high support (100, 100, 1); (8) nine species of *Habenaria* (p.p.) with high support (100, 100, 1); (9) two species of *Gennaria* (100, 100, 1); (10) six species of *Satyrium* (100, 100, 1). The ML and BI nrITS trees are a better resolved than that from MP (Additional file 2: Figure S2).

The maximum parsimony analysis of nrITS (excluding the species that, exhibit topological incongruence between data sets) retrieved 30 MPTs with a tree length of 7241 steps, CI = 0.20, and RI = 0.79. The strict consensus tree (Additional file 3: Figure S3) shows a similar topology as Additional file 2: Figure S2, with the exception of the six species excluded due to their incongruent positions.

The maximum parsimony analysis of nrITS + *Xdh* (excluding nrITS of incongruent species) retrieved 240 MPTs with a score of 9082, CI = 0.26, and (RI) = 0.80. The strict consensus tree (Additional file 4: Figure S4) shows a similar topology (Additional file 2: Figure S2) except for the position of the six incongruent species. These six species are here nested within the clade formed by *Ponerorchis* and *Hemipilia* (Additional file 4: Figure S4).

### Trees inferred from combined molecular data

**Fig. 2:**
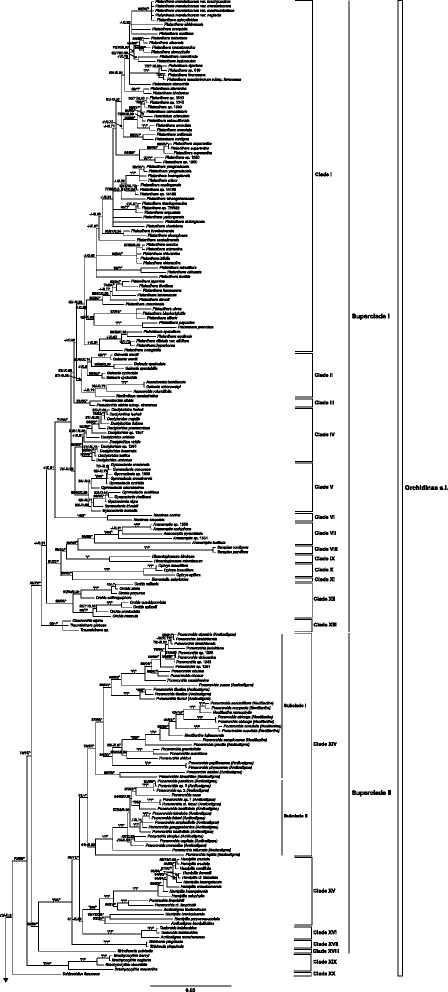
Tree of Orchidinae s.l. from Bayesian inference based on the combined nuclear ITS (excluding ITS of six conflict species), *Xdh*, and five plastid markers. Numbers above branches indicate bootstrap percentages (BS) for ML and MP analyses and posterior probabilities (PP) for BI analysis, respectively. A dash (−) indicates support at a node < 50%, Asterisk (*) indicates BS = 100 or PP = 1.0

**Fig. 3:**
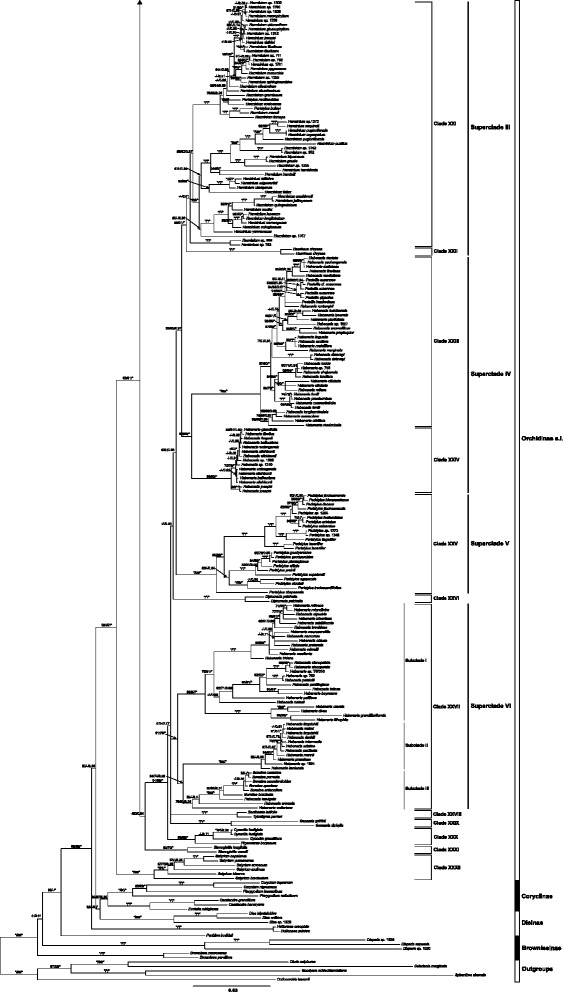
Continuation of the tree in Fig. 2

The MP analysis retrieved 120 MPTs with a tree length of 18,051, CI = 0.34, and RI = 0.81. The strict consensus tree (Figs. 2, 3) shows that Orchidinae can be divided into 32 strongly or moderately supported clades along the backbone. Inter-relationships among most of these clades are well resolved. The ML tree inferred from the combined data has a similar topology to the MP strict consensus tree (Figs. 2, 3; Additional file 4: Figure S4). The BI topology from the combined dataset is presented as the tree for the discussion of phylogenetic relationships (Figs. 2, 3) because it is better resolved and supported.

Clade I comprises 74 species of *Platanthera* s.l. with high or weak support (55, < 50, 0.98; Fig. 2)*.* Clade II consists of nine species of *Galearis* s.l. (in which we including three monotypic genera formerly recognized, *Aceratorchis, Amerorchis* Hultén and *Neolindleya*) with high to weak support (67, < 50, 0.98; Fig. 2). Clade III includes two species of *Pseudorchis* Ség. with high to weak support (81, 65, 1) (Fig. 2). Clade IV consists of 11 species of *Dactylorhiza* (90, 89, 1; Fig. 2). Clade V includes ten species of *Gymnadenia* R.Br., and, like its sister clade IV, is strongly supported (95, 96, 0.99). It is subdivided into two weakly support subclades, one of which corresponds to the formerly recognized genus *Nigritella* Rich. The sister group relationship of clades IV and V is highly to moderately supported (73, 59, 0.99) (Fig. 2). Clade VI consists of two species of *Neotinea* Rchb.f. sister to the group formed by clades I–V with weak support < 50, < 50,  0.51,) (Fig. 2)*.* Strongly supported clade VII consists of eight species of *Anacamptis* Rich., and is sister with strong support to the likewise strongly supported clade VIII made up of two species of *Serapias* (99, 100, 1). Clade IX consists of two species *Himantoglossum* (L.) W.D.J.Koch with strong support (100, 100, 1); it is sister to the group formed by clades VII and VIII with strong support. Clade X (*Ophrys*) and clade XI (*Steveniella* Schltr.) are sister groups with strong (ML + BI) or weak (MP) support (93, 1, 68). Clade XII consists of seven species of *Orchis* s.s. with strong support (95, 96, 1), subdivided into two well supported subclades. Clade XIII is formed by *Chamorchis* Rich. and *Traunsteinera* Rchb. with weak to strong support (64, 50, 1). These 13 clades form the moderately supported superclade I (86, 72, 1).

Clade XIV includes 44 species of *Ponerorchis* s.l. with strong to weak support (91, < 50, 1) and is subdivided into two moderately to strongly supported subclades: subclade I includes *Neottianthe*, *Ponerorchis* s.s. and some species of *Amitostigma* (79, 67, 1), including the type species of all three genera; subclade II includes most of the species formerly assigned to *Amitostigma* (64, < 50, 0.99) (Fig. 2). Six taxa from Japan, i.e. *Amitostigma keiskei* (Finet) Schltr., *A. kinoshitae* (Makino) Schltr., *A. lepidum* (Rchb.f.) Schltr.*, Ponerorchis chidori* (Makino) Ohwi, *P. graminifolia* Rchb.f., and *P.* [*graminifolia* var.] *suzukiana* (Ohwi) J.M.H.Shaw*,* formed a strongly supported clade in the nrITS phylogram, sister to the clade formed by *Hemipilia + Amitostigma + Ponerorchis + Tsaiorchis*. However, these same species have widely different phylogenetic positions in the *Xdh* + plastid trees (Additional file 4: Figure S4). Our analyses suggest that this hard incongruence needs more sampling and research to explain (see discussion below).

Clade XV is also strongly supported and consists only of *Hemipilia*, including a few species previously assigned to *Amitostigma* and *Ponerorchis* (100, 99, 1). Clade XVI includes three species of *Tsaiorchis* Tang & F.T.Wang with strong support (100, 100, 1). Clade XVII only includes the recently established monotypic genus *Shizhenia* X.H.Jin, L.Q.Huang, W.T.Jin & X.G.Xiang (Fig. 1a), which is sister to the moderately to strongly supported clades XIV–XVI (83, 71, 1). Clade XVIII only includes *Sirindhornia* H.A.Pedersen & Suksathan. These last five clades (XIV–XVIII) form the strongly supported superclade II (99, 88, 1). Clade XIX (just *Brachycorythis*) was strongly supported as sister to superclades I + II with moderate to strong support (70, 66, 1), whereas clade XX, consisting of *Schizochilus* Sond.*,* is sister to clades I–XIX with weak support (72, < 50, 0.6).

Clades I–XX and the following clades XXI–XXXI are two major, but highly supported sister groups (93, 81, 1), corresponding to the split between Orchidinae s.s. and Habenariinae in other classifications (e.g. [3, 24]).

The weakly supported clade XXI (< 50, 52, 0.81) consists of *Herminium* s.l., subdivided into six subclades that have moderate to strong support. Clade XXII includes two samples of *Hsenhsua* (formerly in *Ponerorchis*) sister to *Herminium*. These two clades form superclade III with high support (95, 91, 1).

Clade XXIII includes six species of *Pecteilis* Raf. and 45 species of *Habenaria* with strong support (100, 99, 1), subdivided into two well-supported groups: (i) four species from tropical Asia (*H. longicorniculata*–*H. rhodocheila*) and (ii) species from both tropical and subtropical Asia (*Pecteilis* and species of *Habenaria*). *Habenaria delavayi* Finet is sister to a strongly to moderately supported (97, 55, 1) group of 20 species, within which *Pecteilis* is deeply nested. Three yellow-flowered species, including *H. linguella* Lindl., are sister to the white-flowered *H. marginata* Colebr. Clade XXIV, consisting entirely of *Habenaria* species, includes a poorly resolved group formed by ten species. *Habenaria josephi* Rchb.f. is sister to the remainder of this group. Sister clades XXIII and XXIV with strong support (90, 90, 1) form superclade IV.

Strongly supported (100, 99, 1.00) clade XXV is formed by 19 species of *Peristylus*. Clade XXV (= superclade V) is sister to superclades III + IV with weak to strong support (62, 50, 0.96). Strongly supported clade XXVI (100, 100, 1) consists of two samples of *Diplomeris* D. Don, which are sister to superclades III–V, with weak support (< 50, < 50, 0.63).

Clade XXVII consists of *Bonatea* Willd. and 40 species of *Habenaria*, including *H. macroceratitis* Willd. (the type of the genus) with strong support (91, 79, 1). It is subdivided into three well-supported groups (Fig. 3). Subclade I is the core *Habenaria* group comprising *Habenaria rolfeana*–*H. lithophila* (99, 97, 1). This group, which includes *H. macroceratitis*, is characterized by bilobed petals (except for *H. petitiana* (A.Rich.) T. Durand & Schinz), consists of species from tropical Africa, Asia and South America. *Habenaria rolfeana–H. tridens* are entirely American, and no other *Habenaria* species in our dataset are from the Americas. Subclade II comprises *H. limprichtii*–*H. keniensis* (100, 96, 1). This is a group of (sub)tropical to subalpine African and Asian species, which is characterized by the pectinate lateral lobes of the lip, and entire petals (Fig. 1d). Weakly to well- supported (76, 63, 0.95) Subclade III includes *Bonatea* and some African species of *Habenaria*. Clade XXVII forms superclade VI.

Clade XXVIII includes *Benthamia* Rich. (one species) and *Tylostigma* Schltr. (one species) with strong support (100, 100, 1). Clade XXIX includes *Gennaria* Parl. (two species) with strong support (100, 100, 1). The last three clades form a polytomy in all three analyses.

Clade XXX includes *Cynorkis* Thouars (three species) and *Physoceras* Schltr. (one species) with strong support (99, 99, 1). Clade XXXI includes two species of *Stenoglottis* Lindl. with strong support (89, 86, 1). Clades XXX and XXXI are resolved as successive sisters to the group formed by superclades III–VI and clades XXVIII and XXIX. Clade XXXII includes six species of *Satyrium* with strong support (100, 99, 1) and is sister to the rest of Orchidinae with strong support (99, 97, 1).

## Discussion

### Phylogenetics and distribution of Orchidinae s.l.

With broad sampling across Orchidinae and seven DNA markers, many new or previous overlooked phylogenetic relationships were discovered in our analyses. Orchidinae s.l. (comprising Orchidinae s.s., Habenariinae and Satyriinae of other classifications, e.g. [26]) are monophyletic with strong support and divided into three main clades: *Satyrium* (Africa and Asia)*,* Orchidinae s.s. (mainly Northern Hemisphere), and the formerly recognised Habenariinae’ (Fig. 3; nearly cosmopolitan). *Satyrium* is sister to the rest of Orchidinae s.l. with strong support.

Orchidinae s.s. include two superclades and two smaller clades sister to these. Superclade I has high to moderate support and includes 12 sampled genera: *Anacamptis*, *Chamorchis*, *Dactylorhiza*, *Galearis*, *Gymnadenia*, *Himantoglossum, Neotinea, Ophrys, Orchis* s.s*.*, *Platanthera*, *Steveniella,* and *Traunsteinera.* Most of these genera with ovoid/ellipsoid/globose tubers have their center of diversity in Europe and the Mediterranean region, but the two genera with palmately lobed tubers, i.e., *Dactylorhiza* and *Gymnadenia*, extend into the Far East (*Dactylorhiza* into North America), whereas two genera with rhizomes or fleshy rootstocks, i.e., *Galearis* and *Platanthera*, have two centers of diversity, the Pan-Himalayan region and North America.

Superclade II (97, 88, 1), according to our delimitation (discussed below), includes five genera: *Hemipilia*, *Ponerorchis, Shizhenia, Sirindhornia*, and *Tsaiorchis*. This is a subtropical to temperate clade with ovoid/ellipsoid/globose tubers and basal leaf (leaves) occurring almost exclusively in eastern Asia and the eastern Himalayas, with a single species (*Ponerorchis cucullata* (L.) X.H. Jin, Schuit. & W.T.Jin) occurring as far west as Poland. In the subtropical zone the members of this superclade is normally found above 1000 m asl., often in alpine regions [4, 31].

Two relatively isolated genera, *Schizochilus* and *Brachycorythis*, were resolved as successive sister to these two superclades with weak to strong support (Fig. 2). *Schizochilus* is exclusively from eastern to southern Africa, whereas *Brachycorythis* is most diverse in tropical and southern Africa, but also has some species in tropical Asia as far east as Taiwan. Unlike many other Orchidinae, *Brachycorythis* does not occur at alpine elevations in Asia [32]; it is essentially a tropical montane genus.

The clade formerly referred to as Habenariinae (Clades XXI–XXXI) include four superclades (III–VI) and four isolated clades. Chase et al. (2003; 2015) decided not to recognise Habenariinae because of uncertainty over phylogenetic placements of genera such as *Androcorys* (synonym of *Herminium*, see Raskoti (2016)), *Brachycorythis* and *Holothrix*, and until all of the missing genera of Orchidinae s.l. are included, it would be premature to resurrect Habenariinae and not necessary regardless of the final phylogenetic outcome. Superclade III comprises the genera *Herminium* and *Hsenhsua* and is exclusively Asian, except for a single taxon that extends into Europe (*H. monorchis* (L.) R.Br. ex Aiton). Most are alpine plants, including the species that probably holds the elevational record in Orchidaceae (*H. pugioniforme* Lindl. ex Hook.f. at c. 5200 m). Superclade IV includes *Pecteilis* (often included in *Habenaria*) and about 50 species of *Habenaria* mainly distributed in tropical Asia. Superclade V includes only one genus, *Peristylus,* sister to superclades III + IV with weak support. *Peristylus* is only found in tropical Asia, from the lowlands to the alpine zone. The small tropical Asian genus *Diplomeris* is sister to these three superclades with weak support. Superclade VI includes *Bonatea* and 42 species of *Habenaria*; this superclade is distributed in the Americas, tropical and southern Africa, and tropical Asia. A number of small clades are well supported, but their relationships with other Habenariinae are still largely unresolved. *Benthamia* and *Tylostylis* are two sister genera from Madagascar, comprising clade XXVIII, whereas clade XXIX contains the two species of *Gennaria*, one from the Canary Islands and the other from the Himalayan region, a remarkable disjunction. Clade XXX contains two genera from Madagascar and the Mascarenes, *Cynorkis* (also in southern Africa) and *Physoceras* and is resolved as sister to former Habenariinae with strong support. The African genus *Stenoglottis*, often included in Habenarrinae, is sister to the remainder of Habenariinae, but with weak support.

### Phylogeny and generic delimitation

Usually there are many ways to translate a phylogenetic tree into a Linnean classification. In the interest of nomenclatural stability it is desirable that a classification is broadly accepted. One way to help ensure such acceptance is by using objective criteria for generic delimitation as far as possible. Bateman, as cited in Tang et al. (2015), proposed the following five guidelines:

1.   Recognize only monophyletic groups (clades);
2.   Preferentially divide the tree at branches that are relatively robust;
3.   Preferentially divide the tree at branches that receive similar levels of statistical support (obviously, there exists considerable overlap between Rules 2 and 3);
4.   Minimize the proportion of branches in the tree that represent more than one taxonomic rank (notably monotypic higher taxa);
5.   Preferentially divide the tree in a way that minimizes the need (a) to create new names and/or (b) to create new combinations of existing names.

Although we would agree to a large extent with the utility of these guidelines (always allowing for exceptions), we think that one consideration is missing: there is no reference to morphology. We would suggest that a classification supported by morphology is likely to gain acceptance more readily than one based solely on molecular criteria. Therefore, we propose the following addition:

(6)   Preferentially divide the tree in such a way that the points of division correspond to morphological discontinuities (in particular those representing evolutionary innovations).

Ultimately, we are mainly interested in the topology of our phylogenetic tree. Measures of internal support inform us of clarity in the phylogenetic signal of the data analysed. Morphological criteria should also be applied; they not only help us reduce the large number of potential classification schemes based on a given tree, but also make the resulting classification of greater practical value, and thereby easier to accept. However, without a formal cladistics analysis of morphological data, such applications of morphological criteria are ad hoc and likely to be subjective and ignore conflicting characters while emphasising those preferred by one set of authors. Below we discuss a number of troublesome alliances in which consensus has not yet been reached, as well as others in which we are able to present a consensus agreed by most authors.

### *Bonatea, Diplomeris, Habenaria, Herminium, Pecteilis*, and *Peristylus*

Morphologically, *Diplomeris* is an isolated genus in Orchidinae, characterized by its large, white flowers with an entire lip, a column with well developed, semi-circular connective about two thirds the length of the column, two elongate, oblong stigmatic lobes extending from near the entrance of the spur, and a large, shield-like midlobe of the rostellum. Ecologically, it is unusual in Orchidinae in that the species grow as lithophytes on cliffs with seasonal running water trickling down the moss-covered rock. This genus of two species is mainly distributed in tropical to subtropical regions in the Himalayas [2, 4, 33] and would be difficult to place without molecular data. It is somewhat unexpected that *Diplomeris* is sister to the group formed by superclades III–V, but support for this position is as yet weak only.

*Peristylus* consists of about 70 species mainly distributed in tropical Asia [2, 34]. It is not always easy to distinguish from *Peristylus* from *Habenaria* and *Herminium* [2, 10, 23, 33–35]. Recent studies have demonstrated that most alpine species of *Peristylus* actually belong to *Herminium* [10, 23]. With the exclusion of these species, *Peristylus* is distributed mainly in the lowland and montane zones of tropical and subtropical Asia. Our results indicate that, excluding the misplaced species, *Peristylus* is monophyletic and characterized by its elliptic tuber, more or less clearly trilobed and spurred lip, narrow connective, and two stigma lobes adnate to the entrance of the spur. The species of *Peristylus* analysed are distributed over four strongly supported clades: (1) *P. chapaensis* in subtropical limestone regions; (2) a tropical group, including *P. stocksii* (Hook.f.) Kraenzl.; (3) a subtropical montane subclade including *P. affinis*; (4) another subtropical montane subclade including *P. densus* (Lindl.) Santapau & Kapadia. Each subclade is to a large extent characterized by minor morphological characters, especially of the spur and lip.

*Pecteilis* consists of about eight species distributed in southern and eastern Asia and the Malay Archipelago [4, 30, 31]. Morphologically, it is similar to *Habenaria*, but differs in its concave, sessile stigma lobes, unlike the stalked stigmaphores of *Habenaria* [2, 30]. We find that *Pecteilis* is not monophyletic and deeply nested within clade XXIII (otherwise containing species currently accepted as members of *Habenaria* by most authors), and forms a strongly supported subclade interspersed with species of *Habenaria*. It seems that the stigmaphore has been lost independently at least twice in this subclade. Except for the stigmaphore, several members of *Habenaria* of clade XXIII share morphological characters with *Pecteilis*, such as an anther with a broad connective, thecae extending from base of column, and large, subrhombic to flabellate lateral lobes of the lip (Fig. 1h). On the other hand, many other species have linear to filiform lateral lobes, such as *H. plurifoliata* Tang & F.T.Wang, and *H. propinquior* Rchb.f. Species in clade XXIV, which most recent authors include in *Habenaria*, are mostly from alpine habitats in Asia (especially in Himalayan region), and are characterized by two basal, almost opposite leaves appressed to the ground, lateral lobes of the lip linear to filiform, green to white flowers, and petals often more or less distinctly bilobed. Some species in clade XXIII also have leaves appressed to the ground, such as *H. roxburghii* D.H.Nicolson and *H. delavayi* (the latter also with linear lateral lobes to the lip, but usually with three or more leaves in a rosette). On the other hand, the petal lobes in clade XXIV are sometimes indistinct, making the two clades ultimately difficult to tell apart. Together they form superclade IV.

Our results indicate that *Habenaria* is polyphyletic but with low support; it is divided into two well supported but not immediately related groups, superclades IV and VI. Superclade IV, discussed above under *Pecteilis*, is exclusively Asian, and is sister to superclade III (*Herminium* and *Hsenhsua*). A sister-group relationship of superclade VI with any other clade in Habenariinae (Fig. 2) has not been established, since superclade VI consisting of all African and southern American taxa sampled so far as well as some Asian ones is part of a polytomy.

There are at least two divergent options to treat the polyphyly of *Habenaria*. One option would be to expand the delimitation of *Habenaria* to include *Benthamia*, *Bonatea*, *Diplomeris*, *Gennaria, Herminium*, *Hsenhsua*, *Pecteilis*, and *Tylostigma* (Clades XXI–XXIX), many of which have been in the past considered members of *Habenaria* (e.g., many species of *Benthamia, Bonatea*, *Gennaria*, *Herminium*, *Pecteilis*, *Tyolostigma* have combinations in *Habenaria*). *Herminium* would be the earliest available name for this assemblage. Therefore, ironically, an attempt to keep all the species of *Habenaria* within a single genus would lead to the loss of the name *Habenaria*, unless it was conserved. This option would cause minimal nomenclatural upheaval, and the resulting genus could be diagnosed by the characters used to delimit the formerly recognised Habenariinae. A perhaps more disruptive option would be to split *Habenaria* into at least two genera, a decision that would likely result in no agreement over the number of segregate genera and how to recognise them.

The main problem with recognising two genera, the two larger clades into species of *Habenaria* fall, is that there appear to be no consistent morphological differences between the two superclades. However, perhaps it is possible to circumscribe the two groups in a more circuitous way, based on polythetic sets. For example, bilobed petals are, as far as we can tell, only found within certain subclades (in both superclades, unfortunately); similarly, pectinate or laciniate lateral lobes of the lip (Fig. 1d) only occur in some branches of our tree. When the group has been more fully studied it is perhaps possible to identify the two superclades using multiple sets of character states that are unique to each of the two.

Splitting *Habenaria* into more than two genera is another option to be considered. We note, however, that many character states show much homoplasy, and suspect that a more finely split classification will not be easier to use. Instead of having two difficult-to-define genera, we may end up with five or more difficult-to-define genera. This would only increase the potential for misidentifications, without having any obvious benefits, but some authors D. Szlachetko and collaborations have already started down this route, describing new genera based on only morphological characters, mostly column details, which typically results in polyphyletic circumscriptions (such as [36–38]). At this stage it would be premature to propose new combinations.

### *Brachycorythis, Hemipilia, Ponerorchis, Tsaiorchis, Sirindhornia* (superclade II)

*Brachycorythis*, consisting of 30–35 species, is mainly distributed in Africa, with a few species in Asia. It is characterized by leaf-like floral bracts and a more or less bipartite, spurred lip. Recent molecular studies indicate that *Brachycorythis* is sister to the rest of Orchidinae s.s. [23, 25, 26], and that *Sirindhornia* is sister to the clade formed by *Ponerorchis*, *Hemipilia* and related genera [23]. Both findings are also supported by our results, in which *Sirindhornia* is sister to clades XIV–XVII. Tang et al. [24] inferred (but with only moderate support) that *Brachycorythis* is sister to *Hemipilia* s.l. (in which these authors include *Ponerorchis*, *Amitostigma*, *Neottianthe* and *Tsaiorchis*), and that *Sirindhornia* is sister to the remaining Orchidinae s.s. We have been unable to replicate this result in our phylogenetic analyses. Morphologically, *Brachycorythis* is less similar morphologically to the *Hemipilia* alliance than *Sirindhornia*.

Tang et al. [24] detected a hard incongruence involving a group of six taxa (*Ponerorchis graminifolia*, *P.* [*graminifolia* var.] *suzukiana*, *P. chidori*, *Amitostigma lepidum*, *A. keiskei*, and *A. kinoshitae*) between nrITS and four plastid markers and stated that this most likely reflected plastid capture. They concluded that their ITS results were more likely to reflect the actual phylogenetic relationships. Our results indicate that, based on plastid markers, these six taxa, which are endemic to Japan, are deeply nested within clade XIV, but in different positions in the tree. *Amitostigma keiskei* and *A. kinoshitae* are sister to the subclade formed of *Ponerorchis alpestris*–*P. physoceras* (Fig. 2). *Amitostigma lepidum* is sister to a subclade formed exclusively of *Amitostigma* (s.l.) species: *A. parciflora*–*A.trifurcata*. The remaining three species (including *Ponerorchis graminifolia*) are sister to the subclade formed of *Neottianthe* species + *Amitostigma gracile* (Blume) Schltr. Based on nrITS alone, according to the results of Tang et al. [24] and our analyses of nrITS (Additional file 2: Figure S2), these six species form a strongly supported clade sister to our clades XIV–XVI. Since they include the type species of *Ponerorchis* (*P. graminifolia*), this would imply, as Tang et al. [24] correctly pointed out, that our clade XIV could not be called *Ponerorchis*, as it would not include the type species of *Ponerorchis*.

Morphologically, these six species do not form a uniform group, and the scattered placement of its members in the plastid tree more accurately reflects their diverse morphology than their occurrence in a single clade. We consider it unlikely that six taxa that are all endemic to Japan and morphologically disparate, happen to form a single clade of which the members have somehow integrated the plastid genomes of at least three different, now extinct progenitors that are closely related to geographically isolated, extant Chinese taxa.

Based on our results from nuclear markers (ITS +*Xdh*) we infer that the six taxa are nested within a polytomy of *Ponerorchis* s.l., which is sister to *Hemipilia* + *Tsaiorchis* with moderate support (BS_ML_ = 78, BS_MP_ = 67, PP = 0.72) (Additional file 4: Figure S4). They do not form a clade, and their placement does not render *Ponerorchis* in the sense of Jin et al. [23] poly- or paraphyletic.

It remains an interesting problem to explain how ITS data could produce such discordant results in the case of these six taxa. It is well known that certain molecular genetic processes involving ITS, such as ancient or recent array duplication events, genomic harbouring of pseudogenes, and incomplete intra- or inter-array homogenization, may distort results of phylogenetic inference and need to be examined [39]. Whatever the cause for the incongruence, on morphological grounds and the plastid and combined nuclear molecular results, we prefer to continue to recognize *Hemipilia, Ponerorchis* and *Tsaiorchis* as distinct genera, while adding *Shizhenia*, which is sister to the three others combined.

### *Platanthera*

Although most species-rich in Asia, *Platanthera* is also diverse in North America, and a few segregate genera have been recognized there (e.g. *Blephariglottis* Raf., *Limnorchis* Rydb., *Piperia* Rydb., and others). In a recent revision of the Asian taxa [20], the genus was taken in the broad sense, which corresponds with our clade I.

*Platanthera* is among the largest genera of the tribe Orchideae, consisting of some 130 species, and is mainly distributed in Asia and North America, with a few species in Europe and North Africa [2, 19]. Historically, the generic delimitation and phylogenetics of *Platanthera* were confusing [19, 21, 28, 40]. Recent results indicate that once a few misplaced species are transferred to the proper genera, *Platanthera* s.l. is monophyletic and relatively well characterized by morphology, such as fleshy, more or less tapering roots, often forming fusiform tuberoids, a spurred lip, a short and truncate column, and a broad connective [19, 23, 41] (Fig. 1c).

Efimov [20] recognized five subgenera, which were recovered in our tree. The *Platanthera minor* group comprises about eight species distributed in subtropical regions in East Asia; it is characterized by several cauline leaves attenuating into bracts, a dense inflorescence, and a narrowly elliptic lip narrowed at the base. This group in subgenus *Platanthera* could not be assigned to a section by Efimov [20]. The *Platanthera minutiflora* group displays disjunction between East Asia and North America. It was recognized by Efimov [20] as subgen. *Platanthera* sect. *Lysiella* (Rydberg) Efimov. The *Platanthera carnosilabris* group (99, 100, 1) in subgen. *Platanthera* includes about eight species that are characterized by small flowers, a lip more or less concave at base, and spur shorter than the lip. Efimov [20] could not place these in a section. This group includes the misplaced *Herminium orbiculare* Hook.f. Moderate support (75, 75, 0.97) is shown for subgen. *Platanthera* sect. *Stigmatosae* (Lang) Efimov (*P. bakeriana–P. leptocaulon*).

### *Stenoglottis, Schizochilus*

Both *Stenoglottis* and *Schizochilus* are small genera endemic to southern Africa. The phylogenetic positions of *Stenoglottis* and *Schizochilus* have not been fully established in previous studies, and are still uncertain. Bateman et al. [26] resolved *Stenoglottis* as sister to Habenariinae s.s. Inda et al. [25], on the other hand, found *Stenoglottis* to be sister to the rest of their tribe Orchideae (our Orchidinae s.l.). Our own results show weak support for the position inferred by Bateman et al. (< 50, 60, 0.64).

*Schizochilus* is here found to be sister to Orchidinae s.s., but with weak support (72, < 50, 0.6). Morphologically, *Schizochilus* is similar to *Brachycorythis* and *Sirindhornia*, especially in column morphology.

### *Satyrium* (Clade XXXII)

*Satyrium* comprises about 90 species and is mainly distributed in temperate and montane regions of Sub-Saharan Africa, with a few species occurring in Asia, mainly in the Himalayan region [2]. Based on its distinctive morphology, including non-resupinate flowers, a galeate lip with two spurs, stalked column, and pad-like stigmatic lobes, which sets it apart from other Orchidinae, *Satyrium* has been treated as a monotypic subtribe Satyriinae [2, 42]. Published molecular studies indicate that *Satyrium* is monophyletic and sister to Orchidinae s.l. [25, 26, 43, 44], which is also strongly supported by our results 99, 97, 1; Fig. 2). Chase et al. [1] assigned *Satyrium* to Orchidinae, which was motivated especially by the desire to minimize the number of monogeneric higher taxa. This is also in agreement with guideline 4 above (chapter 4.2). Considering the distinctive morphology of *Satyrium*, a case could be made to reinstate Satyriinae, but similar arguments could be advanced for many other subtribes. We would prefer not to increase the complexity of our classification if this can be avoided. Limiting the number of monogeneric higher taxa as far as possible is a means towards this end. In addition, it would appear from the work of Inda et al. [25] that the genus *Holothrix* is sister to *Satyrium* + the remainder of Orchidinae s.l. Therefore, recognition of a subtribe Satyriinae would also require the recognition of another subtribe for *Holothrix* (which might include other, related genera that have not been analysed yet). It is certainly simpler to keep them all within Orchidinae.

### *Gennaria* (Clade XXIX)

There are two species in *Gennaria*, the type species *G. diphylla* Parl. in the Mediterranean region plus the Canary Islands, the other (the recently transferred *G. griffthii* (Hook.f.) X.H.Jin., L.Q.Quang, W.T.Jin & X.G.Xiang, Fig. 1e) in the Pan-Himalayan region [2, 45]. Morphologically, *Gennaria* is characterized by subcampanulate flowers more or less secund along the rachis, a short column with stalked lateral appendages, and convex stigmas [2, 46]. Inda et al. [25] found *Gennaria* to be deeply nested within *Habenaria*. Batista et al. [9] had *Gennaria* as sister to *Habenaria + Bonatea*, but with low support, which is similar to the analysis of Jin et al. (2014). Our new results show that *Gennaria* is a strongly supported clade that does not nest within *Habenaria*. Instead, it is one of the branches of a polytomy formed otherwise by superclade VI, Clade 26, and Clade 25. It may be an outlier in this alliance, which is also suggested by the extremely wide but fragmented distribution area of the clade, with a low number of extant species.

## Conclusions

With increased understanding of the phylogenetics of Orchidinae s.l. a stable classification system of generic level is close to being reached. The main outstanding problem is the apparent polyphyly of the largest genus in this alliance, *Habenaria*. Another area of contention is in the *Ponerorchis*-*Hemipilia* alliance, where a hard conflict exists between results from ITS and those from plastid + *Xdh* analyses. *Satyrium* is best included within a broader Orchidinae rather than in its own monotypic subtribe Satyriinae, especially since its exclusion would probably require that a separate subtribe is also needed for *Holothrix*. Future studies will undoubtedly shed light on the geographical origin of the various clades and the timing of their divergence.

## Methods

### Taxon sampling, DNA extraction, amplification and sequencing

We sampled 436 accessions representing approximately 400 species in 52 genera of Orchidinae (Additional file 5: Table S1). Five species from tribes Cranichideae and Diurideae were used as outgroups based on previous results [1, 47, 48].

Total genomic DNA was isolated from silica-gel-dried tissue using a Plant Genomic DNA Kit (Tiangen Biotech, LTD, Beijing, China) following the manufacturer’s protocols and a modified CTAB method [49]. For this study, seven markers including two nuclear markers (nrITS and *Xdh*) and five plastid markers (*matK*, *psaB*, *psbA-trnH*, *rbcL*, and *trnL-F*) were used. Amplification of all DNA regions was performed in 25 μL reactions containing 10–50 ng DNA, 12.5 μL 2× Taq PCR Master Mix (Biomed, Beijing, China), 0.4 μM of each primer, and ddH_2_O to reach a total volume of 25 μL. The PCR profiles used in the current study are presented in Additional file 6: Table S2. The sequencing reactions were performed with the ABI Prism Bigdye Terminator Cycle Sequencing Kit (Applied Biosystems, ABI). Amplification and sequencing primers are listed in Additional file 6: Table S3.

### Phylogenetic analyses

Sequences were edited independently and assembled using the ContigExpress program in Vector NTI Suite v. 6.0 (Informax, North Bethesda, Maryland, USA). The edited sequences were aligned using MAFFT v. 7.221 [50] with auto strategy and default settings. The aligned sequences were then manually adjusted using BioEdit v. 7.25 [51]. Ambiguously aligned characters in the *psbA-trnH* and *trnL-F* datasets were excluded before phylogenetic analyses.

Congruence between nuclear (nrITS and *Xdh*) and plastid datasets (*matK*, *psaB*, *psbA-trnH*, *rbcL*, and *trnL-F*) was tested using the incongruence length difference (ILD) [52] implemented in PAUP v4.0b10 [53]. The ILD test used an analysis of 100 replicates, with random addition sequence, TBR swapping, and no more than 15 trees of score (length) greater than or equal to 5 were saved in each replicate. The *P*-value was 0.01 in this study, and regarded as significant incongruence following Cunningham (1997). In the present study, the incongruence between data sets was also assessed by a comparing the topologies and support of the trees produced by the data partitions. The thresholds of hard incongruence here followed previous work [54]: (1) ILD *P* < 0.01; (2) bootstrap percentages ≥ 80 and/or posterior probabilities (PP) ≥ 95. These tests indicated a hard incongruence between nrITS topologies on the one hand and plastid, *Xdh,* plastid *+ Xdh* topologies on the other. When the nrITS dataset of these six conflicting taxa was removed from the combined matrix, the results (see results) did not indicate hard incongruence. Therefore, we combined the nuclear and plastid datasets in SequenceMatrix v. 1.7.8 [55] to perform further phylogenetic analyses, but with the nrITS dataset of these six taxa excluded from the combined matrix.

Phylogenetic analyses were performed for each matrix using maximum likelihood (ML), maximum parsimony (MP) and Bayesian inference (BI). ML inference relied on RAxML-HPC2 v. 8.2.4 [56] on XSEDE via the CIPRES Science Gateway online web server [57]. The ML analysis used the GTRCAT nucleotide substitution model following other default settings and ran 1000 rapid bootstrap replicates to obtain the best-scoring ML tree with support percentages. The MP analyses were performed in PAUP v. 4.0b10 (Swofford, 2002). All characters were equally weighted, and gaps were coded as missing data. Heuristic searches of 1000 random addition replicates with tree bisection reconnection (TBR) branch swapping were conducted to obtain the most parsimonious trees; 10 trees were held in each iteration step of the TBR-swapping procedure. To evaluate the node support, bootstrap analyses [58] were performed using 500 replicates with 10 random taxon additions and heuristic search. For BI analysis, posterior probabilities (PP) for individual clades were inferred with MrBayes v. 3.2.3 [59] on XSEDE via the CIPRES Science Gateway online web server [57]. The best-fitting DNA substitution model for each matrix was determined using MrModeltest v. 2.3 [60] under the Akaike information criterion (AIC). Two separate four-Markov-chain Monte Carlo (MCMC) analyses were performed, starting with a random tree, proceeding for 10,000,000 generations, and sampling every 1000 generations. The tree or posterior probabilities was constructed after discarding the burn-in phase samples (the first 25% of sampled trees).

## Additional files

Additional file 1: Figure S1. Tree of Orchidinae s.l. from Bayesian inference based on plastid markers. Numbers above branches indicate bootstrap percentages (BS) for ML and MP analyses and posterior probabilities (PP) for BI analysis, respectively. The dash (−) indicates support at a node < 50%, Asterisk (*) indicates BS = 100 or PP = 1.0. (PDF 427 kb)

Additional file 2: Figure S2. Tree of Orchidinae s.l. from Bayesian inference based on nrITS, including ITS of six species for which position conflict between the plastid and nrITS results. Numbers above branches indicate bootstrap percentages (BS) for ML and MP analyses and posterior probabilities (PP) for BI analysis, respectively. The dash (−) indicates support at a node < 50%, and an asterisk (*) indicates BS = 100 or PP = 1.0. (PDF 438 kb)

Additional file 3: Figure S3. Tree of Orchidinae s.l. from Bayesian inference based on ITS, excluding nrITS of the six species with incongruent positions. Numbers above branches indicate bootstrap percentages (BS) for ML and MP analyses and posterior probabilities (PP) for BI analysis, respectively. A dash (−) indicates support at a node < 50%, and an asterisk (*) indicates BS = 100 or PP = 1.0. (PDF 435 kb)

Additional file 4: Figure S4. Tree of Orchidinae s.l. from Bayesian inference based on nrITS and Xdh, excluding the nrITS sequences of the six species with incongruent positions. Numbers above branches indicate bootstrap percentages (BS) for ML and MP analyses and posterior probabilities (PP) for BI analysis, respectively. A dash (−) indicates support at a node < 50%, and an asterisk (*) indicates BS = 100 or PP = 1.0. (PDF 439 kb)

Additional file 5: Table S1. Voucher information and GenBank accession numbers for the sequences analysed in this study, *indicates newly produced sequences for this paper. The dash (−) indicates the missing sequences. (XLSX 46 kb)

Additional file 6: Table S2. The PCR programs used for amplifying the DNA regions. **Table S3.** List of primers used for PCR and sequencing in this study. **Table S4.** Statistics from analyses of nuclear and plastid datasets. (DOCX 21 kb)

## Abbreviations

AIC: Akaike information criterion
BI: Bayesian inference
ILD: Incongruence length difference
MCMC: Markov-chain Monte Carlo
ML: Maximum likelihood
ML_BS_: Bootstrap values of maximum likelihood
MP: Maximum parsimony
MP_BS_: Bootstrap values of maximum parsimony
PP: Posterior probabilities
