# Supplementary figures and images for: Phylogenetics of subtribe Orchidinae s.l. (Orchidaceae; Orchidoideae) based on seven markers (plastid matK, psaB, rbcL, trnL-F, trnH-psba, and nuclear nrITS, Xdh): implications for generic delimitation
Source: BMC Plant Biol. 2017 Nov 25;17:222. doi: 10.1186/s12870-017-1160-x (PMC5702240; doi:10.1186/s12870-017-1160-x)

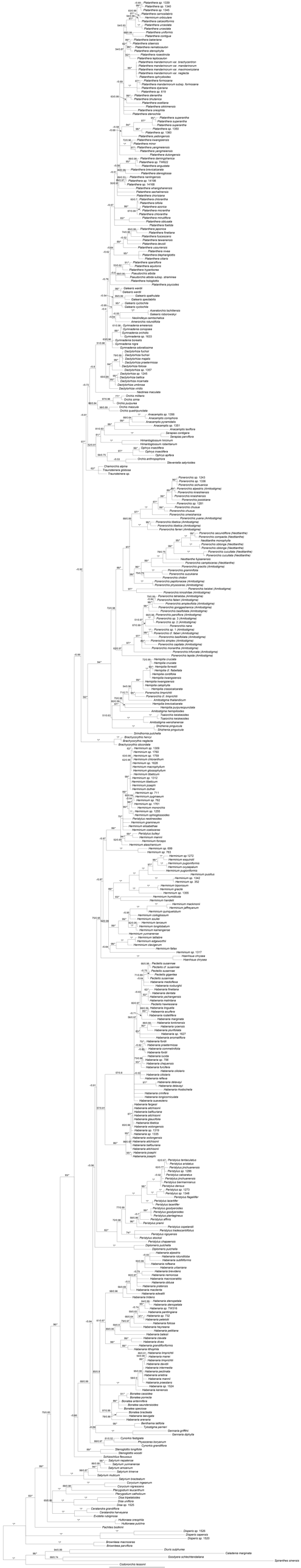

Supplement: Supplementary file 1 — Tree of Orchidinae s.l. from Bayesian inference based on plastid markers. Numbers above branches indicate bootstrap percentages (BS) for ML and MP analyses and posterior probabilities (PP) for BI analysis, respectively. The dash (−) indicates support at a node < 50%, Asterisk (*) indicates BS = 100 or PP = 1.0. (PDF 427 kb) [file 12870_2017_1160_MOESM1_ESM.pdf]

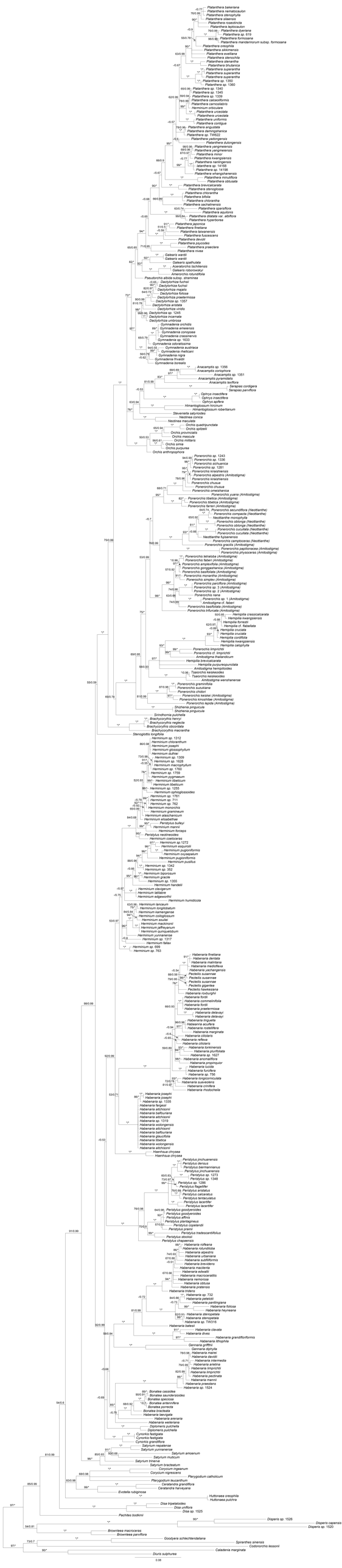

Supplement: Supplementary file 2 — Tree of Orchidinae s.l. from Bayesian inference based on nrITS, including ITS of six species for which position conflict between the plastid and nrITS results. Numbers above branches indicate bootstrap percentages (BS) for ML and MP analyses and posterior probabilities (PP) for BI analysis, respectively. The dash (−) indicates support at a node < 50%, and an asterisk (*) indicates BS = 100 or PP = 1.0. (PDF 438 kb) [file 12870_2017_1160_MOESM2_ESM.pdf]

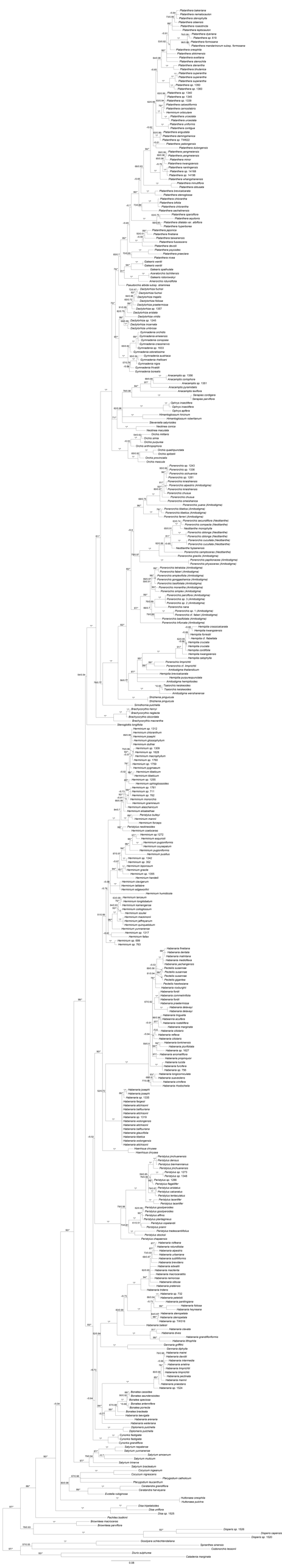

Supplement: Supplementary file 3 — Tree of Orchidinae s.l. from Bayesian inference based on ITS, excluding nrITS of the six species with incongruent positions. Numbers above branches indicate bootstrap percentages (BS) for ML and MP analyses and posterior probabilities (PP) for BI analysis, respectively. A dash (−) indicates support at a node < 50%, and an asterisk (*) indicates BS = 100 or PP = 1.0. (PDF 435 kb) [file 12870_2017_1160_MOESM3_ESM.pdf]

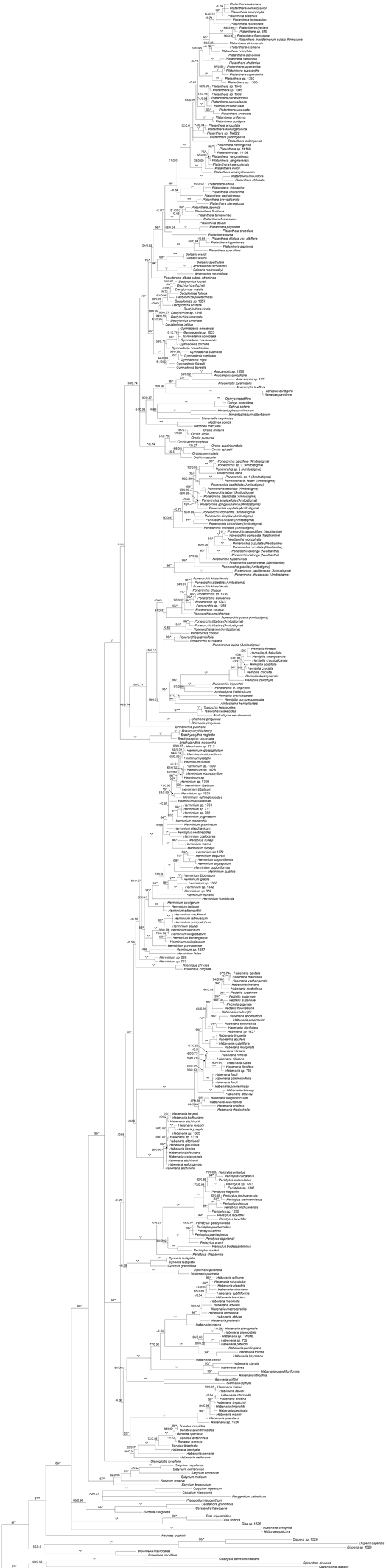

Supplement: Supplementary file 4 — Tree of Orchidinae s.l. from Bayesian inference based on nrITS and Xdh, excluding the nrITS sequences of the six species with incongruent positions. Numbers above branches indicate bootstrap percentages (BS) for ML and MP analyses and posterior probabilities (PP) for BI analysis, respectively. A dash (−) indicates support at a node < 50%, and an asterisk (*) indicates BS = 100 or PP = 1.0. (PDF 439 kb) [file 12870_2017_1160_MOESM4_ESM.pdf]
