# Supplementary material for: Phylogenetics of subtribe Orchidinae s.l. (Orchidaceae; Orchidoideae) based on seven markers (plastid matK, psaB, rbcL, trnL-F, trnH-psba, and nuclear nrITS, Xdh): implications for generic delimitation
Source: BMC Plant Biol. 2017 Nov 25;17:222. doi: 10.1186/s12870-017-1160-x (PMC5702240; doi:10.1186/s12870-017-1160-x)
Supplement: Supplementary file 6 — The PCR programs used for amplifying the DNA regions. Table S3. List of primers used for PCR and sequencing in this study. Table S4. Statistics from analyses of nuclear and plastid datasets. (DOCX 21 kb) [file 12870_2017_1160_MOESM6_ESM.docx]

Table S2: The PCR programs used for amplifying the DNA regions in the currenty study

| DNA regions | Pre-melt | Denaturation | Annealing | Extension | No. of cycles | Final extension |
| --- | --- | --- | --- | --- | --- | --- |
| ITS | 94 ℃ (5 min) | 94 ℃ (45 sec) | 55 ℃ (45 sec) | 72 ℃ (60 sec) | 34 | 72 ℃ (10 min) |
| *matK* | 95 ℃ (5 min) | 95 ℃ (30 sec) | 50 ℃ (60 sec ) | 72 ℃ (90 sec) | 34 | 72 ℃ (10 min) |
| *rbcL* | 95 ℃ (5 min) | 95 ℃ (30 sec) | 50 ℃ (60 sec ) | 72 ℃ (90 sec) | 34 | 72 ℃ (10 min) |
| *psaB* | 95 ℃ (5 min) | 95 ℃ (30 sec) | 50 ℃ (60 sec ) | 72 ℃ (90 sec) | 34 | 72 ℃ (10 min) |
| *psbA-trnH* | 94 ℃ (3 min) | 94 ℃ (45 sec) | 52-55 ℃ (60 sec) | 72 ℃ (120 sec) | 34 | 72 ℃ (5 min) |
| *trnL-F* | 94 ℃ (3 min) | 94 ℃ (30 sec) | 50-55 ℃ (30 sec) | 72 ℃ (90 sec) | 34 | 72 ℃ (10 min) |
| *Xdh* | 94 ℃ (2 min) | 94 ℃ (45 sec) | 58 ℃ (45 sec) | 72 ℃ (90 sec) | 34 | 72 ℃ (5 min) |

TableS3: A list of primers used for PCR and sequence in this study.

| Region | Primer | Sequence(5’-3’) | Reference |
| --- | --- | --- | --- |
| ITS | 17SE | ACGAATTCATGGTCCGGTGAAGTGTTCG | Sun et al., 1994 |
|  | 26SE | TAGAATTCCCCGGTTCGCTCGCCGTTAC | Sun et al., 1994 |
|  | ITS-p3 | YGACTCTCGGCAACGGATA | Cheng et al., 2015 |
|  | ITS-p5 | CCTTATCAYTTAGAGGAAGGAG | Cheng et al., 2015 |
|  | ITS-u2 | GCGTTCAAAGAYTCGATGRTTC | Cheng et al., 2015 |
|  | ITS-u4 | RGTTTCTTTTCCTCCGCTTA | Cheng et al., 2015 |
| *matK* | -19F | CGTTCTGACCATATTGCACTATG | Goldman et al., 2001 |
|  | 390F | CGATCTATTCATTCAATATTTC | Cuenoud et al., 2002 |
|  | 1326R | TCTAGCACACGAAAGTCGAAGT | Cuenoud et al., 2002 |
|  | trnK-2R | AACTAGTCGGATGGAGTAG | Johnson & Soltis, 1994 |
| *psaB* | NY159F | ACGCGTCGTATTTGGTTTGGTATTGC | Cameron, 2004 |
|  | NY160R | CAATGCCAATAAAAAGTAACCCATCC | Cameron, 2004 |
|  | NY162R | CCGATATKGCTCATCATCAYTTAGC | Cameron, 2004 |
|  | NY178F | ATGACCAATTCCAAAATTAGTTCTATACAT | Cameron, 2004 |
| *psbA-trnH* | psbA | GTTATGCATGAACGTAATGCTC | Sang et al., 1997 |
|  | trnH2 | CGCGCATGGTGGATTCACAATCC | Tate, 2002 |
| *rbcL* | 1F | ATGTCACCACAAACAGAAAC | Goldman et al., 2001 |
|  | 724R | TGCCATGTACCYGCAGTTGC | Goldman et al., 2001 |
|  | 1360R | CTTCACAAGCAGCAGCTAGTTC | Goldman et al., 2001 |
| *trnL-F* | c | CGAAATCGGTAGACGCTACG | Taberlet et al., 1991 |
|  | d | GGGGATAGAGGGACTTGAAC | Taberlet et al., 1991 |
|  | f | ATTTGAACTGGTGACACGAG | Taberlet et al., 1991 |
| *Xdh* | X502F | TGTGATGTCGATGTATGC | Górniak et al., 2010 |
|  | X551F | GAAGAGCAGATTGAAGAWWGCC | Górniak et al., 2010 |
|  | X1591R | AAYTGGAGCAACTCCACCA | Górniak et al., 2010 |
|  | X1599R | GWGAGAGAAAYTGGAGCAAC | Górniak et al., 2010 |

**References**

Cheng, T., Xu, C., Lei, L., Li, C.H., Zhang, Y., Zhou, S.L., 2015. Barcoding the kingdom Plantae: new PCR primers for ITS regions of plants with improved universality and specificity. Mol. Ecol. Resour. DOI: 10.1111/1755-0998.12438.

Cuénoud, P., Savolainen, V., Chatrou, L.W., Powell, M., Renée, J. Grayer, R.J., Chase, M.W., 2002. Molecular phylogenetics of Caryophyllales based on nuclear 18S rDNA and plastid *rbcL*, *atpB*, and *matK* DNA sequences. Am. J. Bot. 89, 132-144.

Goldman, D.H., Freudenstein, J.V., Kores, P.J., Molvray, M., Jarrell, D.C., Whitten, W.M., Cameron, K.M., Jansen, R.J., Chase, M.W., 2001. Phylogenetics of Arethuseae (Orchidaceae) based on plastid *matK* and *rbcL* sequences. Syst. Bot. 26, 670–695.

Cameron, K.M., 2004. Utility of plastid *psaB* gene sequences for investigating intrafamilial relationships within Orchidaceae. Mol. Phylogenet. Evol. 31, 1157-1180.

Górniak, M., Paun, O., Chase, M.W., 2010. Phylogenetic relationships within Orchidaceae based on a low-copy nuclear coding gene, *Xdh*: Congruence with organellar and nuclear ribosomal DNA results. Mol. Phylogenet. Evol. 56, 784-795.

Johnson, L. A., Douglas, E. Soltis D.E., 1994. *matK* DNA sequences and phylogenetic reconstruction in Saxifragaceae s. str. Syst. Bot. 19, 143-156.

Sang, T., Crawford, D.J., Stuessy, T.F., 1997. Chloroplast DNA phylogeny, reticulate evolution, and biogeography of *Paeonia* (Paeoniaceae). Am. J. Bot. 84, 1120-1136.

Sun, Y., Skinner, D.Z., Liang, G.H., Hulbert S.H., 1994. Phylogenetic analysis of *Sorghum* and related taxa using internal transcribed spacers of nuclear ribosomal DNA. Theor. Appl. Genet. 89, 26–32.

Taberlet, P., Gielly, L., Pautou, G., Bouvet, J., 1991. Universal primers for amplification of three non-coding regions of chloroplast DNA. Plant Mol. Biol. 17, 1105-1109.

Tate, J. A. 2002. Systematics and evolution of *Tarasa* Philippi (Malvaceae): an enigmatic Andean polyploid genus. Ph.D. dissertation. The University of Texas at Austin.

Table S4: The statistics from the analyses of the nuclear and chloroplast datasets.

| Information | ITS | ITS^a^ | *Xdh* | nDNA | *matK* | *psaB* | *psbA-trnH* | *rbcL* | *trnL-F* | cpDNA | Combined |
| --- | --- | --- | --- | --- | --- | --- | --- | --- | --- | --- | --- |
| No. of taxa | 395 | 401 | 275 | 403 | 400 | 265 | 300 | 333 | 311 | 422 | 436 |
| Aligned length | 976 | 977 | 931 | 1907 | 1888 | 1608 | 1372 | 1262 | 1715 | 7845 | 9752 |
| No. of variable characters | 683 | 684 | 541 | 1224 | 1636 | 314 | 465 | 273 | 710 | 2724 | 3948 |
| No. of parsimony-informative characters | 587  (60%) | 586  (60%) | 426  (46%) | 1013  (53%) | 710  (38%) | 203  (13%) | 232  (17%) | 186  (15%) | 469  (27%) | 1800  (23%) | 2813  (29%) |
| Tree length (steps) | 7241 | 7352 | 1756 | 9082 | 3810 | 689 | 1173 | 699 | 2257 | 8819 | 18051 |
| Consistency index (CI) | 0.204 | 0.201 | 0.492 | 0.258 | 0.384 | 0.528 | 0.531 | 0.456 | 0.48 | 0.437 | 0.343 |
| Retention index (RI) | 0.788 | 0.787 | 0.865 | 0.801 | 0.818 | 0.883 | 0.79 | 0.862 | 0.805 | 0.816 | 0.805 |
| Model | GTR+I+G | GTR+I+G | HKY+I+G | GTR+I+G | GTR+I+G | GTR+I+G | GTR+I+G | GTR+I+G | GTR+I+G | GTR+I+G | GTR+I+G |
| No. of excluded ambiguously aligned characters | - | - | - | - | - | - | 270  (16%) | - | 286  (14%) | 556  (7%) | 556  (5%) |

^a^including the six conflict species of *Amitostigma keiskei, Amitostigma kinoshitae, Amitostigma lepidum, Ponerorchis chidori, Ponerorchis graminifolia,* and *Ponerorchis suzukiana* in ITS dataset.
